# Multi-omics Signature of *Candida auris*, an Emerging and Multidrug-Resistant Pathogen

**DOI:** 10.1128/mSystems.00257-19

**Published:** 2019-06-11

**Authors:** Daniel Zamith-Miranda, Heino M. Heyman, Levi G. Cleare, Sneha P. Couvillion, Geremy C. Clair, Erin L. Bredeweg, Attila Gacser, Leonardo Nimrichter, Ernesto S. Nakayasu, Joshua D. Nosanchuk

**Affiliations:** a Department of Microbiology and Immunology, Albert Einstein College of Medicine, Bronx, New York, USA; b Division of Infectious Diseases, Department of Medicine, Albert Einstein College of Medicine, Bronx, New York, USA; c Biological Sciences Division, Pacific Northwest National Laboratory, Richland, Washington, USA; d Environmental and Molecular Sciences Laboratory, Pacific Northwest National Laboratory, Richland, Washington, USA; e Department of Microbiology, Interdisciplinary Excellence Centre, University of Szeged, Szeged, Hungary; f MTA-SZTE “Lendület” Mycobiome Research Group, University of Szeged, Szeged, Hungary; g Instituto de Microbiologia Paulo de Goes, Universidade Federal do Rio de Janeiro, Rio de Janeiro, Brazil; Stanford University

**Keywords:** Candida auris, antifungal resistance, fluconazole, multi-omics

## Abstract

Candida auris is a recently described pathogenic fungus that is causing invasive outbreaks on all continents. The fungus is of high concern given the numbers of multidrug-resistant strains that have been isolated in distinct sites across the globe. The fact that its diagnosis is still problematic suggests that the spreading of the pathogen remains underestimated. Notably, the molecular mechanisms of virulence and antifungal resistance employed by this new species are largely unknown. In the present work, we compared two clinical isolates of C. auris with distinct drug susceptibility profiles and a Candida albicans reference strain using a multi-omics approach. Our results show that, despite the distinct drug resistance profile, both C. auris isolates appear to be very similar, albeit with a few notable differences. However, compared to C. albicans both C. auris isolates have major differences regarding their carbon utilization and downstream lipid and protein content, suggesting a multifactorial mechanism of drug resistance. The molecular profile displayed by C. auris helps to explain the antifungal resistance and virulence phenotypes of this new emerging pathogen.

**IMPORTANCE** Candida auris was first described in Japan in 2009 and has now been the cause of significant outbreaks across the globe. The high number of isolates that are resistant to one or more antifungals, as well as the high mortality rates from patients with bloodstream infections, has attracted the attention of the medical mycology, infectious disease, and public health communities to this pathogenic fungus. In the current work, we performed a broad multi-omics approach on two clinical isolates isolated in New York, the most affected area in the United States and found that the omic profile of C. auris differs significantly from C. albicans. In addition to our insights into C. auris carbon utilization and lipid and protein content, we believe that the availability of these data will enhance our ability to combat this rapidly emerging pathogenic yeast.

## Abstract (precis)

Candida auris was first described in Japan in 2009 and has now been the cause of significant outbreaks across the globe. The high number of isolates that are resistant to one or more antifungals, as well as the high mortality rates from patients with bloodstream infections, has attracted the attention of the medical mycology, infectious disease, and public health communities to this pathogenic fungus. In the current work, we performed a broad multi-omics approach on two clinical isolates isolated in New York, the most affected area in the United States and found that the omic profile of C. auris differs significantly from C. albicans. In addition to our insights into C. auris carbon utilization and lipid and protein content, we believe that the availability of these data will enhance our ability to combat this rapidly emerging pathogenic yeast.

## INTRODUCTION

Candida auris is an emerging pathogenic fungus that was first described in 2009 after being isolated from the ear discharge of a patient in Tokyo, Japan (1). After the new species identification, a study in South Korea reported a misidentified C. auris strain isolated in 1996, which then became the first known case of human C. auris infection (2). Despite the fact that bloodstream infections are the main cause of mortality among *Candida* spp. infections, C. auris has been isolated from various sites, such as the respiratory tract, bones, and central nervous system (3), as well as on a variety of abiotic surfaces (4), which suggests a metabolic plasticity to survive in distinct environments. The reports of C. auris outbreaks in all continents suggest that this pathogen is spreading rapidly across the globe, and many of the isolates are resistant to at least one class of antifungals or even multidrug resistant (5–11). C. auris produces biofilms and can be very resilient in substrates commonly used in hospitals, features that are correlated with the frequency of reported hospital-associated infections, as well as its increased resistance against antifungals (4, 9, 12–15). In addition, its problematic identification suggests that reports regarding infection might be underestimated (16–18).

To understand the molecular mechanisms of infection, antifungal resistance, and disease employed by this new pathogen, we performed a multi-omics approach using two clinical isolates of C. auris and compared to a standard C. albicans strain. The tested C. auris isolates presented different levels of antifungal resistance, since one of them is highly resistant to fluconazole and slightly resistant to caspofungin. Both C. auris isolates had very similar metabolic, lipid, and protein profiles. However, both isolates were significantly distinct compared to C. albicans. Taken together, our data show metabolic, lipidomic, and proteomic similarities and differences between C. auris isolates, as well as in comparison to C. albicans, and our findings provide interesting insights into metabolic features, with some correlating with antifungal resistance.

## RESULTS

### Antifungal resistance.

Since C. auris is a recently identified pathogen, its breakpoints for resistance to different antifungals have not been formally established. Given the lack of information, our results were interpreted based on the Centers for Disease Control and Prevention (CDC) breakpoint suggestions (https://www.cdc.gov/fungal/candida-auris/recommendations.html). The MICs for the tested organisms against amphotericin B were similar, and all of them had an MIC below 2 μg/ml and were thus susceptible to this antifungal. MMC2 was consider susceptible since the MIC to caspofungin was <2 μg/ml. MMC1 had an MIC of 2 μg/ml for caspofungin, which qualifies as resistance to this drug. Notably, C. auris isolates were able to grow when exposed to caspofungin concentrations above their MIC, a phenomenon known as “paradoxical effect” or “Eagle effect” (19). This effect was previously reported for *Aspergillus* and *Candida* species (19) and was very recently described for C. auris (20). C. auris MMC2 was susceptible to fluconazole, presenting an MIC at 8 μg/ml. In contrast, C. auris MMC1 isolate was highly resistant since it was able to grow at concentrations of 1,000 μg/ml of fluconazole (Table 1). As a reference, we also examined a standard C. albicans strain (ATCC 90028), which is susceptible to all the three drugs used in this work.

**TABLE 1:** Antifungal susceptibility test using the broth microdilution

Organism/isolate	MIC (μg/ml)		
	Amphotericin B	Caspofungin	Fluconazole
Candida auris MMC1	1.6	2	>256^*a*^
Candida auris MMC2	0.8	1.6	8
Candida albicans 90028	1.3	0.3	0.75

a MMC1 was resistant to fluconazole concentrations of 1,000 μg/ml.

### Proteomic profiling of *C. auris* versus *C. albicans*.

The proteomic analysis resulted in the identification of 1,869 and 2,317 proteins in C. auris and C. albicans, respectively. To compare the data from these two species, we performed BLAST searches and considered orthologous proteins with >40% similarity. Of the 1,869 identified C. auris proteins, 1,726 (92%) had orthologues in the C. albicans genome, whereas 1,954 of the 2,317 (84%) C. albicans proteins had orthologues in the C. auris genome. In all, 2,323 orthologues were detected in the proteomic analysis. However, only 1,357 (58% of total) orthologues were consistently abundant in both *Candida* species (see Tables S1 to S3 in the supplemental material). This indicates that despite the sequence similarity between these two species, their gene expression regulation is much more divergent even under identical culture conditions.

10.1128/mSystems.00257-19.1 TABLE S1 Proteomic analysis of C. auris isolates. Protein abundances were normalized into relative copies numbers (see Materials and Methods for details). Download Table S1, XLSX file, 0.2 MB. Copyright © 2019 Zamith-Miranda et al. 2019 Zamith-Miranda et al. This content is distributed under the terms of the Creative Commons Attribution 4.0 International license.

10.1128/mSystems.00257-19.2 TABLE S2 Proteomic analysis of C. albicans strain 90028. Protein abundances were normalized into relative copies numbers (see Materials and Methods for details). Download Table S2, XLSX file, 0.2 MB. Copyright © 2019 Zamith-Miranda et al. 2019 Zamith-Miranda et al. This content is distributed under the terms of the Creative Commons Attribution 4.0 International license.

10.1128/mSystems.00257-19.3 TABLE S3 Comparative analysis of C. albicans strain 90028 versus C. auris isolates. Protein abundances were normalized into relative copies numbers. Then values were divided by the average between all samples and transformed into a log_2_ scale (see Materials and Methods for details). Statistically significant comparisons are highlighted in blue, while less and more abundant proteins are highlighted in green and red scales, respectively. Download Table S3, XLSX file, 0.5 MB. Copyright © 2019 Zamith-Miranda et al. 2019 Zamith-Miranda et al. This content is distributed under the terms of the Creative Commons Attribution 4.0 International license.

**FIG 1:**
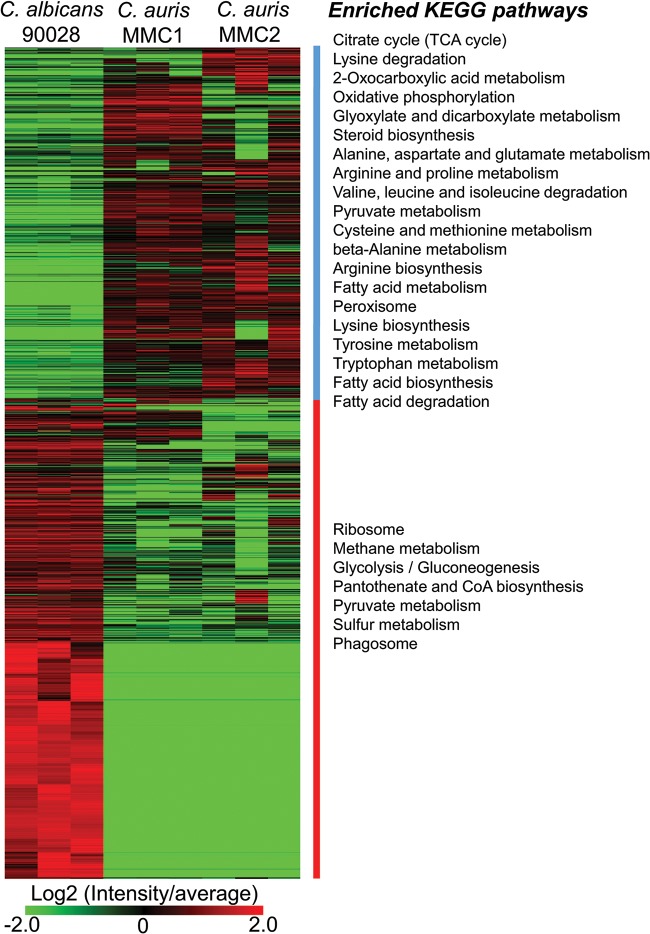
Abundance of proteins in C. auris and C. albicans. Proteins are listed in the heatmap with enriched KEGG pathways separated into two clusters based on the protein abundance between the two *Candida* species. For a complete list of proteins, their relative abundances, and *P* values (determined by *t* test), see Table S3 in the supplemental material.

It is noteworthy that the peptides were not identical between the two species; therefore, a quantitative proteomic analysis comparison cannot be directly achieved across the different samples. To circumvent these issues, we performed an absolute quantification of each protein using the intensity-based absolute quantification (iBAQ) method and normalized each protein by the relative number of copies in the cells. The heatmap shown in Fig. 1 depicts the orthologues that were differentially abundant between both *Candida* species. Clustering these proteins using the k-means method showed a striking similarity between the two C. auris isolates but strong differences between the different species. To better understand the differences between C. auris isolates and also between the *Candida* species, we performed a function-enrichment analysis, which revealed that pathways such as glycolysis/gluconeogenesis, ribosomes, and phagosomes were more abundant in C. albicans. On the other hand, C. auris seemed to have a more active tricarboxylic acid (TCA) cycle, along with lipid and amino acid metabolism.

### Central carbon metabolism in *C. auris* and *C. albicans*.

**FIG 2:**
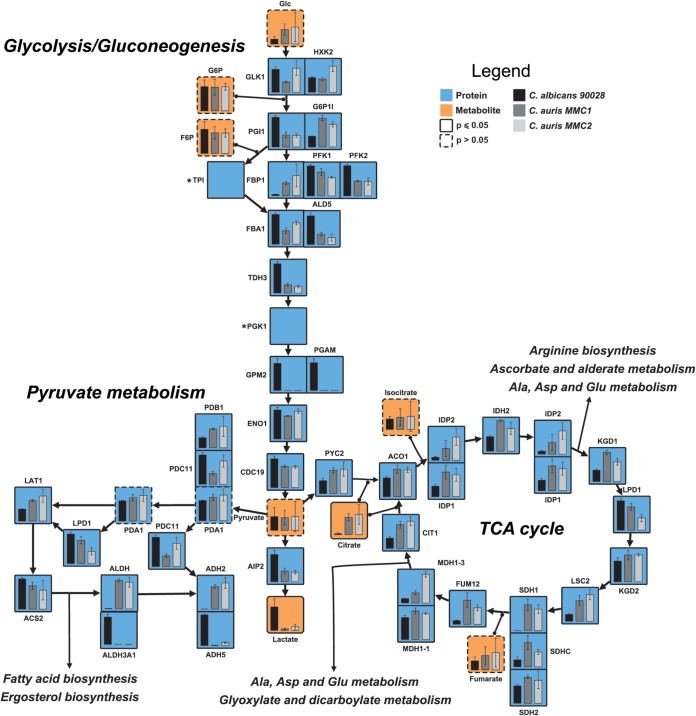
Central carbon metabolism of C. auris and C. albicans. The figure shows the relative abundance of proteins (blue boxes) and the production of metabolites (orange boxes) involved in the central carbon metabolism in both C. albicans and C. auris. Paralog proteins were grouped and posted side by side in the map. *, Genes that were only annotated in the C. albicans genome. *P* ≤ 0.05 indicates statistically significant hits determined by *t* test in any of the three comparisons. For complete comparisons between the different samples and abundances of each analyte, see Table S4 in the supplemental material.

The pathway analysis showed that the glycolytic pathway was enriched in proteins with higher abundance in C. albicans, whereas the TCA cycle proteins were enriched with proteins more abundant in C. auris. Different proteins of the pyruvate metabolism were more abundant in one of the other *Candida* species (Fig. 1). To validate these observations and to correlate with downstream metabolic pathways, we integrated the proteomics data with a metabolite analysis into a map of central carbon metabolism. Ten of the fifteen glycolysis/gluconeogenesis proteins were more abundant in C. albicans than in C. auris, whereas only two proteins were consistently more abundant in C. auris (Fig. 2). In agreement with these observations, lactate, one of the end products of this pathway, was 16-fold more abundant in C. albicans than C. auris MMC1 and 6-fold more abundant in C. auris MMC2 (Fig. 2). On the other hand, 14 of 15 TCA cycle proteins were more abundant in C. auris isolates than in C. albicans (Fig. 2 and Table S4). Further validating these observations, citrate had similar abundance profiles (Fig. 2). In the pyruvate metabolism, proteins were not consistently more abundant in one or the other species. Some differentially abundant proteins seemed to be due to gene isoforms that were preferentially expressed between the species. For example, C. auris produces alcohol dehydrogenase Adh2, while C. albicans produces Adh5 (Fig. 2). Unfortunately, the metabolites of this pathway, such as acetate, acetaldehyde, and ethanol, are small and not detectable in our gas chromatography-mass spectrometry (GC-MS) analysis. The fact that different proteins of this pathway were not uniformly more abundant in one of the species makes it more difficult to predict whether the downstream metabolic pathways would be affected. We decided to investigate the ergosterol and glycerolipid biosynthesis pathways in more detail.

10.1128/mSystems.00257-19.4 TABLE S4 Proteins and metabolites from central carbon metabolism and ergosterol synthesis pathways. Protein abundances were normalized into relative copies numbers. Then values were divided by the average between all samples and transformed into a log_2_ scale (see Materials and Methods for details). Download Table S4, XLSX file, 0.02 MB. Copyright © 2019 Zamith-Miranda et al. 2019 Zamith-Miranda et al. This content is distributed under the terms of the Creative Commons Attribution 4.0 International license.

### Ergosterol biosynthesis pathway in *C. auris* versus *C. albicans.*

**FIG 3:**
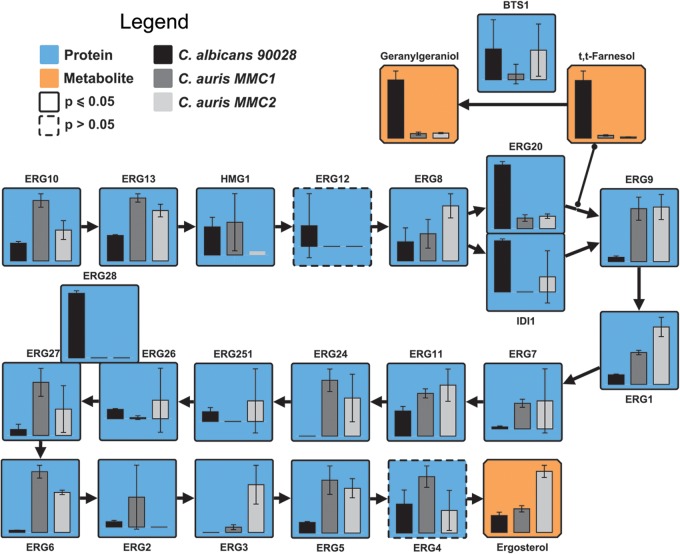
Ergosterol biosynthesis pathway in C. auris and C. albicans. The bar graphs represent the relative abundances of proteins (blue boxes) and metabolites (orange boxes) of the pathway. Note that Erg28 is not an enzyme but a scaffold protein that docs Erg26 and Erg27 close together. *P* ≤ 0.05 indicates statistically significant hits determined by *t* test in any of the three comparisons. For complete comparisons between the different samples and abundances of each analyte, see Table S4 in the supplemental material.

Fluconazole inhibits the activity of Erg11 (lanosterol 14-α-demethylase) and consequently ergosterol biosynthesis. Due to the remarkable resistance displayed by MMC1 against fluconazole, we performed a comparative analysis of the enzymes and some of the metabolites present in the ergosterol synthesis pathway. Eleven (Erg10, Erg13, Erg9, Erg1, Erg7, Erg11, Erg24, Erg27, Erg6, Erg3, and Erg5) of nineteen of the ergosterol synthesis enzymes were significantly more abundant in C. auris MMC1 than in C. albicans, including Erg11 (Fig. 3). Similarly, 7 (Erg13, Erg8, Erg9, Erg1Erg6, Erg3, and Erg5) of 13 of the ergosterol synthesis enzymes were significantly more abundant in C. auris MMC2 than in C. albicans, including Erg11 (Fig. 3). Ergosterol itself was four times more abundant in MMC2 compared to MMC1 and C. albicans and had similar abundances in MMC1 and C. albicans (Fig. 3). There were, however, a few exceptions of proteins from the ergosterol pathway that were more abundant in C. albicans than in C. auris, which was the case for Idi1, Erg20, and Erg28. Idi1 and Erg20 seem to diverge from the pathway to produce farnesol, a quorum-sensing molecule involved in C. albicans dimorphism and its downstream product geranylgeraniol. Farnesol was 20.7- and 51.8-fold more abundant in C. albicans compared to C. auris MMC1 and MMC2, respectively (Fig. 3). Similarly, geranylgeraniol was 12.9- and 11.6-fold times more abundant in C. albicans compared to C. auris MMC1 and MMC2, respectively (Fig. 3). Erg28 is a scaffold protein that docs Erg26 and Erg27 close together (21), but how its abundance affect enzymatic reaction still needs be to investigated.

### Lipid profile of *C. auris* and *C. albicans.*

**FIG 4:**
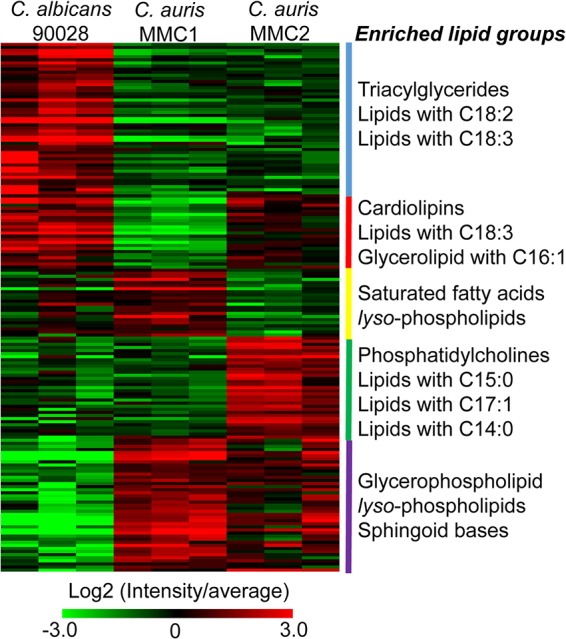
Lipid species found in C. auris and C. albicans. The abundance of all detected lipids is shown above in the heatmap. Lipids were grouped in clusters based on their abundance between different species/isolates. The enrichment of lipid intrinsic features (head group, fatty acid length, fatty acid unsaturation, etc.) is listed by the side of each cluster. For a complete list of proteins, their relative abundances, and *P* values (determined by *t* test), see Table S5 in the supplemental material.

The differential abundance of carbon metabolism, especially in the pyruvate metabolism, is indicative that the fatty acid (FA) biosynthesis and consequently the lipid structures could be altered. Considering that lipids are major targets of antifungal drugs (22) and part of resistance mechanisms (23, 24), we analyzed this category of biomolecules. A total of 169 lipids from 10 different classes were identified and quantified. The most diverse lipid class was triacylglycerol (TG), with 38 distinct species, followed by phosphatidylcholine (PC) with 28 (Table S5). To compare groups of lipids from different *Candida* species/isolates, we clustered lipids based on their abundance and performed an enrichment analysis using a recently developed tool named Lipid Mini-On (described in Materials and Methods). This analysis is analogous to pathway enrichment and determines whether groups of lipids are significantly enriched based on their intrinsic features (class, head group, FA length, and unsaturation, etc.). The results showed that TG and lipids carrying polyunsaturated FA were enriched in C. albicans. Cardiolipins, lipids containing C_18:3_ FA and glycerolipids carrying C_16:1_ FA were significantly reduced in the resistant isolate MMC1 (Fig. 4). Lysophospholipids were enhanced in C. auris MMC1 and to a lesser extent in C. auris MMC2 compared to C. albicans. The enriched amount of lysophospholipids is an indication of a higher phospholipase activity. We investigated the abundance profiles of enzymes with phospholipase activity in the proteomics data (Table 2). Our analysis detected seven phospholipases in C. auris and only five in C. albicans. Excepting Pld1 (A0A0L0P056), all of them were significantly by *t* test more abundant in MMC1 than in C. albicans. Remarkably, lysophospholipases Plb3 (A0A0L0NWB3) and Plb5 (A0A0L0P465) were not detected in C. albicans.

**TABLE 2:** Proteins with phospholipase activity in C. auris and C. albicans

Protein	C. auris UniProt no.	Relative abundance^*a*^		
		C. albicans 90028	C. auris MMC1	C. auris MMC2
Plc2p	A0A0L0P5S6	−	+ +	+
Patatin-like phospholipase domain-containing protein	A0A0L0NS42	− −	+ +	−
Lysophospholipase	A0A0L0NWB3	ND	+ +	+ +
	A0A0L0P465	ND	+	+ +
Doa1p	A0A0L0NP71	+	+ +	+ +
Phospholipase	A0A0L0P056	+	+	+
Lysophospholipase Nte1 (intracellular phospholipase B)	A0A0L0P1C1	+ +	−	−

a +, <0.5 and >−1; + +, >0.5; –, >−3 and <−1; – –, <−3; ND, not determined.

10.1128/mSystems.00257-19.5 TABLE S5 Comparative lipidomic analysis of C. albicans strain 90028 versus C. auris isolates. Lipid intensities were divided by the average between all samples and transformed into a log_2_ scale (see Materials and Methods for details). Statistically significant comparisons are highlighted in blue, while less and more abundant lipids are highlighted in green and red scales, respectively. Download Table S5, XLSX file, 0.06 MB. Copyright © 2019 Zamith-Miranda et al. 2019 Zamith-Miranda et al. This content is distributed under the terms of the Creative Commons Attribution 4.0 International license.

**FIG 5:**
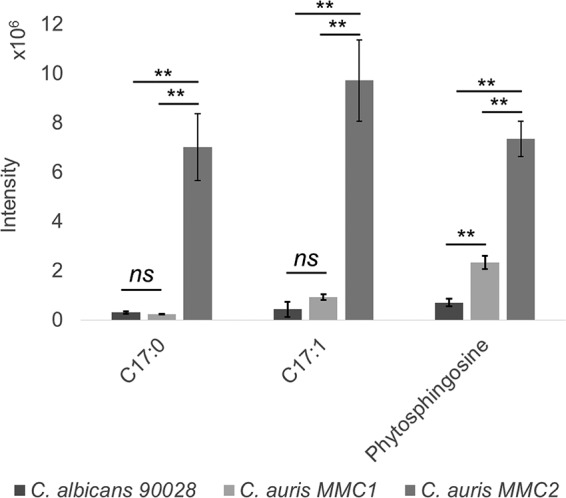
Fatty acids and sphingoid bases analyzed by GC-MS. The graph indicates the abundance of lipids containing odd-chain fatty acids and phytosphingosine for both *Candida* species/isolates. *t* test determinations: **, *P* ≤ 0.01; ns, *P* > 0.05.

C. auris MMC2 produced more phosphatidylcholines and lipids containing odd-chain FA compared to C. auris MMC1 and C. albicans (Fig. 4). A GC-MS analysis of the lipid fraction indeed confirmed that C_17:0_ and C_17:1_ FA were more abundant in C. auris MMC2 (Fig. 5). C_17:0_ was 23.3- and 28.9-fold more abundant in C. auris MMC2 compared to C. albicans and C. auris MMC1, respectively. Similarly, C_17:1_ was 22.3- and 10.5-fold more abundant in C. auris MMC2 compared to C. albicans and C. auris MMC1, respectively (Fig. 5). Both isolates of C. auris were enriched in sphingoid bases (Fig. 4), which was also validated by the detection of phytosphingosine in the GC-MS analysis. This sphingolipid was 3.3-fold more abundant in C. auris MMC1 compared to C. albicans and 10.3- and 3.2-fold more abundant in C. auris MMC2 compared to C. albicans and C. auris MMC1, respectively (Fig. 5). In addition to the sphingoid bases, other sphingolipids such as ceramides, hexosylceramides, and inositolphosphoceramides were also more abundant in C. auris MMC1 (Table S5).

### Cell wall integrity pathway and major structural components.

**FIG 6:**
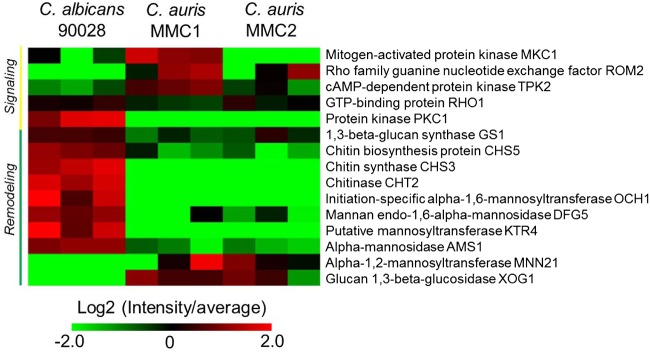
Cell wall integrity pathway. The heatmap includes signaling and major cell wall polysaccharides synthesis/degradation enzymes found in C. auris and C. albicans. For a complete list of proteins, their relative abundances, and *P* values (determined by *t* test), see Table S6 in the supplemental material.

The proteomic analysis showed that proteins involved in the cell wall integrity (CWI) pathway displayed a significant difference between C. albicans and C. auris. Rom2, Tpk2, and the mitogen-activated protein kinase Mck1 were higher in MMC1 compared to C. albicans and the fluconazole-susceptible MMC2 (Fig. 6), suggesting that the MMC1 isolate is better suited to respond to this antifungal. Notably, the protein Pkc1 was detected only in C. albicans, suggesting that C. auris may have an alternative pathway to control CWI (Fig. 6).

10.1128/mSystems.00257-19.6 TABLE S6 Comparative analysis of proteins from C. albicans strain 90028 and C. auris isolates involved with biofilm. Protein abundances were normalized into relative copies numbers. Then values were divided by the average between all samples and transformed into a log_2_ scale (see Materials and Methods for details). Download Table S6, XLSX file, 0.01 MB. Copyright © 2019 Zamith-Miranda et al. 2019 Zamith-Miranda et al. This content is distributed under the terms of the Creative Commons Attribution 4.0 International license.

The enzymes involved in the synthesis and degradation of the major cell wall polysaccharides (glucans and chitin) and mannoproteins were particularly distinct when C. albicans and C. auris were compared. Remarkably, the chitin remodeling enzymes, β1,3-glucan synthase, and most of the mannoprotein remodeling enzymes were higher in C. albicans compared to both C. auris isolates. The only exceptions were glucan 1,3-β-glucosidase Xog1 and α-1,2-mannosyltransferase MN21, which were both more abundant in C. auris isolates compared to C. albicans (Fig. 6).

### Biofilm transcription factors and proteins.

Fungal biofilms are highly resistant to drug treatment due to a combination of factors, including cell density and matrix content (25). We compared the abundance of transcription factors and proteins previously reported in biofilm formation and proteins found in the biofilm matrix. Six transcription factors were reported as biofilm regulators in C. albicans (26–29). Our results showed that Efg1 and Ndt80 were more abundant in C. albicans under planktonic growth conditions with almost no abundance in C. auris. Remarkably, only Rob1 was more abundant in C. auris, specifically in the resistant isolate MMC1. A list of proteins upregulated in C. albicans biofilms and biofilm matrix was also investigated (Table S6). Of 24 proteins previously reported upregulated in biofilm (30), 8 were detected at higher levels in the C. auris isolates than in C. albicans.

### Transporters.

**FIG 7:**
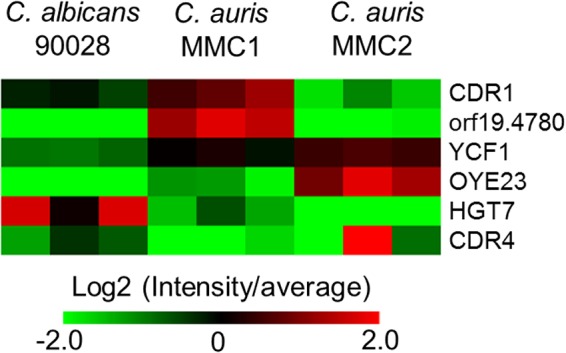
Protein abundance profile of drug resistance-related transporters. The heatmap shows the detected transporters involved with drug resistance and their abundances in both *Candida* species/isolates. For a complete list of proteins, their relative abundances, and *P* values (determined by *t* test), see Table S3 in the supplemental material.

The proteomic analysis identified six transporters related to drug resistance. Notably, the ABC transporter efflux pump Cdr1 and orf19.4780, an uncharacterized member of the Dha1 family of drug:proton drug antiporter, were significantly higher in the azole-resistant isolate MMC1 (Fig. 7). The other four transporters showed greater abundance in either MMC2 or C. albicans (Fig. 7); therefore, they are less likely to be involved in the fluconazole resistance of MMC1.

## DISCUSSION

C. auris is an emerging pathogen that is causing extremely worrisome outbreaks across the globe. One remarkable feature of this fungus is the frequency of resistance against at least one class of antifungals. In addition, multidrug-resistant strains have been isolated from all continents. The search for a new class of antifungal drug has been a major challenge in the medical mycology community, and this quest becomes even more urgent with the spread of a multidrug-resistant fungal organism such as C. auris. In the present study, two clinical isolates of C. auris isolated in the Bronx, NY, were analyzed by a multi-omics approach to better understand the molecular repertoire employed by this pathogen. In parallel to C. auris, we also performed the same analyses with a reference strain of C. albicans. C. auris has been classified into four distinct clades according to their biogeography (31). A recent epidemiological study by the CDC (32) has shown that the vast majority of C. auris isolates from New York City belong to the South Asian clade (98% of all analyzed isolates). Even though there are obvious limitations about studying a small number of isolates, our study was nevertheless able to delve deep into the C. auris biology and provides a platform for future analyses of additional isolates.

We found that MMC2 and the C. albicans strain were susceptible to amphotericin B, caspofungin, and fluconazole, but MMC1 was resistant to both caspofungin and fluconazole. The C. auris MMC2 MIC value of fluconazole was approximately 8 μg/ml, which, based on the CDC report, would make it a susceptible isolate, even though the MIC was about 10 times higher than for C. albicans. Although MMC1 just met resistance criteria to caspofungin, its resistance to fluconazole was impressive, as even 1 mg/ml was not able to totally inhibit growth. The “Eagle effect,” also known as “paradoxical effect” was observed in both C. auris isolates after treatment with caspofungin, since growth occurred at concentrations higher than the MIC.

The protein profiles from C. auris and C. albicans were qualitatively and quantitatively distinct, and both isolates of C. auris presented very few differences from one another (Table S3). The major observed difference between C. auris and C. albicans was in their central carbon metabolism. While proteins in the glycolysis pathway were upregulated in C. albicans, C. auris showed an enrichment of proteins in the TCA cycle. These results show that C. auris favors respiration, which is already known to be an important mechanism of fluconazole resistance in C. albicans, by increasing ATP production and reducing oxidative stress, resulting in better overall fitness of the cell (33).

In Saccharomyces cerevisiae, overexpression of HMG1 or deletion of ERG2, can significantly increase susceptibility to fluconazole, whereas the deletion of HMG1, ERG6, and ERG3, as well as the overexpression of ERG11, is associated with fluconazole resistance (34). Therefore, we integrated the data of proteins and metabolites of the ergosterol biosynthesis pathway. Despite the extreme resistance of MMC1 against fluconazole, the abundance of Erg11 in this isolate was similar to that observed for MMC2. On the other hand, the higher abundance of Erg2 and lower abundance of Erg3 of MMC1 compared to the MMC2 isolate are in agreement with drug resistance phenotype of MMC1. The higher abundance of Idi1 and Erg20 in C. albicans diverges part of the pathway to produce more isoprenoids, while C. auris has a more robust production of ergosterol, which is possibly involved in fluconazole resistance. Recently, sequence divergences/mutations on ERG11 in C. auris have been shown to be associated with resistance to azoles (35). However, the ERG11 mutations by themselves cannot explain why the level of fluconazole resistance was lower (up to 128 μg/ml) when the C. auris gene was expressed in S. cerevisiae (36). Therefore, our data combined with reports from the literature suggest that the fluconazole resistance in C. auris is due to modifications of multiple steps in the ergosterol biosynthesis pathway.

The lipids detected in C. auris were qualitatively similar to those found in C. albicans. However, a quantitative analysis showed that C. albicans has more lipids involved with energy storage, while C. auris has more structural glycerophospholipids and lysophospholipids. The resistant isolate (MMC1) has a remarkable abundance of lysophospholipids, suggesting intense phospholipase activity. Phospholipases are virulence factors in a variety of pathogenic fungi where their activity is important for invasiveness, morphology, and persistence of infection (37–39). Phospholipase activity was recently described in C. auris isolates (40). In the present study, the evaluated C. auris isolates were found to produce seven enzymes with phospholipase activity, while C. albicans had five of them. In addition, most of these enzymes were more abundant in C. auris, particularly in the resistance isolate (MMC1). Corroborating these findings, an increased content of lysophospholipids was previously reported in a C. albicans strain adapted *in vitro* to higher concentrations of fluconazole (41). It is possible that this class of enzymes is more finely employed by C. auris than by C. albicans to promote survival and environmental adaptation for the fungus. Regarding its biological role during the host-pathogen interaction, lysophosphatidylcholine is a “find me” signal released by apoptotic cells to induce the recruitment of phagocytes to remove apoptotic bodies before an episode of secondary necrosis and enhanced inflammation (42). The MMC1 isolate also had a higher abundance of sphingolipids, which can also be correlated with resistance to antifungals. These lipids are important for the assembly of membrane platforms where proteins such as drug efflux pumps are present in membrane microenvironments responsible for the export of drugs (23).

The response orchestrated by the CWI signaling pathway is central during cell wall and membrane perturbation (43). Sensors at fungal cell surface initiate a downstream cascade in order to adapt the cells under stress conditions controlling cell wall biogenesis and cell integrity (43). Remarkably, we observed that the enzymes involved with cell wall remodeling were reduced in both C. auris isolates. However, some CWI proteins were specifically higher in the resistant isolate, suggesting that the response to external signals, such as drug treatment, could be promptly controlled by the cell wall metabolism and help to explain the resistant phenotype in the MMC1 isolate.

The efflux of drugs mediated by efflux pumps is an important mechanism of antifungal resistance employed by *Candida* spp. (23, 44, 45). Of six distinct drug efflux transporters produced by the analyzed organisms, two (CDR1 and orf19.4780) were more abundant in the fluconazole-resistant C. auris isolate (MMC1) than in MMC2 or C. albicans. Previous publications showed that C. auris yeast cells, organized in a biofilm, are more resistant to antifungals than planktonic cells and correlated this phenotype with the increased expression of CDR1 (13). The impact of these efflux pumps is important during early stages of biofilm formation but decreases when it becomes mature. In mature biofilms, resistance is increased by the ability of matrix components to limit drug diffusion, along with the presence of persistent cells (46). Notably, the C. auris isolate MMC1 has a significant increase in proteins associated with biofilm formation and a higher abundance of superoxide dismutase, an enzyme involved with reactive oxygen species (ROS) detoxification and overexpressed in miconazole-tolerant persisters (46). Furthermore, several proteins characterized in the biofilm matrix were also higher in the resistant C. auris isolate.

The comprehensive multi-omics approach used in this study has enabled us to begin to uncover and characterize the molecular profile of the emerging pathogen C. auris, which suggest a multifactorial mechanism of drug resistance in MMC1, including major differences in carbon utilization, sphingolipids, glycerolipids, sterols, the cell wall, and efflux pumps. Further functional omic studies that include larger numbers of C. auris isolates will likely have significant impact on our understanding of the biology of this remarkable fungus and may facilitate the development of new therapeutic approaches to combat this frequently multidrug-resistant yeast.

## MATERIALS AND METHODS

### Cell lines.

Two clinical isolates (MMC1 and MMC2) were acquired from Montefiore Medical Center (Bronx, NY) under approved protocols in the Nosanchuk laboratory, and a standard C. albicans (ATCC 90028) strain was purchased from the American Type Culture Collection (ATCC). The cells were stored at –80°C. Prior to use in experiments, cells were cultivated in yeast extract-peptone-dextrose (YPD) broth and seeded onto Sabouraud agar plates. For each experiment, one colony was inoculated in 10 ml of Sabouraud broth overnight at 30°C before use. Cells were transferred to 200 ml of fresh Sabouraud medium and incubated for an additional 24 h. After being extensively washed with phosphate-buffered saline (PBS), the cell pellets were frozen until the protein, metabolite, and lipid extractions.

### Antifungal susceptibility.

The antifungal susceptibility tests were carried out based on the CLSI protocol with modifications (47, 48). Yeast cells were inoculated in Sabouraud agar for 48 h at 30°C and then stored at 4°C up to 1 month for experimentation. One colony from each organism was inoculated in Sabouraud broth and kept for 24 h at 30°C under constant shaking. Cells were then washed in PBS and plated (2.5 × 10^3^ cells/ml) in 96-well plates containing serial dilutions of amphotericin B, caspofungin, and fluconazole. After 48 h of incubation in Sabouraud broth in the presence or absence of antifungals, cells were visually analyzed, and the MIC was determined as the lowest concentration of a given drug that showed no apparent growth within all replicates.

### Proteomic analysis.

Three independent cell cultures were submitted to metabolite, protein, and lipid extraction (MPLEx) according to the protocol by Nakayasu et al. (49). Extracted proteins were digested with trypsin, and the resulting peptides were extracted with 1 ml of Discovery C_18_ SPE columns (Supelco, Bellefonte, PA) as previously described (50). Digested peptides were suspended in water, quantified by BCA assay and 0.5 μg of peptides were loaded into trap column (4 cm by 100 μm inner diameter [ID], packed in-house with 5 μm C_18_; Jupiter). Peptide separation was carried out an analytical column (70 cm x 75 μm ID packed with C_18_, 3-μm particles) using a gradient of acetonitrile–0.1% formic acid (solvent B) in water–0.1% formic acid (solvent A). The flow was set to 300 nl/min with 1% solvent B and kept for 15 min. Then, the concentration of solvent B was increased linearly as follows: 19 min, 8% B; 60 min, 12% B; 155 min, 35% B; 203 min, 60% B; 210 min, 75% B; 215 min, 95% B; 220 min, 95% B. Eluting peptides were directly analyzed by electrospray in an orbitrap mass spectrometer (Q-Exactive Plus; Thermo Fisher Scientific) by scanning a window of 400 to 2,000 *m/z* with a resolution of 70,000 at *m/z* 400. Tandem mass spectra were collected using high-energy collision dissociation (32% normalized collision energy) on the 12 most intense multiple-charged parent ions at a resolution of 17,500.

Mass spectrometry data were analyzed using MaxQuant software (v.1.5.5.1) (51). Peptide identification was performed by searching against the C. albicans SC5314 and C. auris sequences from Uniprot Knowledge Base (downloaded 6 December 2017). The search parameters included the variable modifications protein N-terminal acetylation and oxidation of methionine, in addition to carbamidomethylation of cysteine residues. Parent and fragment mass tolerance were kept as the default setting of the software. Only fully tryptic digested peptides were considered, allowing up to two missed cleaved sites per peptide. Quantification of proteins was done using the intensity-based absolute quantification (iBAQ) method (52). Intensities of each protein were normalized by the total iBAQ sum of each sample to obtain a relative protein copy number (percentage from total). The comparison between the two species was performed by blast searches and considering a cutoff of 40% of sequence similarity to consider a protein orthologous.

### Lipid analysis.

Extracted lipids were suspended in 100% methanol and analyzed by liquid chromatography-tandem mass spectrometry (LC-MS/MS) as described elsewhere (53). The identification of the species was done using LIQUID software and manually inspected for validation (54). Peak intensities of each identified lipid species were extracted with MZmine v2.0 (55).

### Gas chromatography-mass spectrometry analysis.

Extracted hydrophilic metabolite and lipid fractions were derivatized as described previously (56) and analyzed in an Agilent GC 7890A using an HP-5MS column (30 m × 0.25 mm × 0.25 μm; Agilent Technologies, Santa Clara, CA) coupled with a single quadrupole MSD 5975C (Agilent Technologies). The GC was set to splitless mode with the port temperature at 250°C. Samples were injected with the oven temperature equilibrated at 60°C. The same temperature was kept for 1 min and then raised at a 10°C/min rate to a final temperature of 325°C for a 5-min hold. A standard mixture of fatty acid methyl ester (Sigma-Aldrich) was used for calibrating the retention time. Retention time calibration, spectral deconvolution, and peak alignment were done with MetaboliteDetector (57). Metabolites were identified by matching against FiehnLib library (58) containing additional metabolites entered in-house and/or the NIST14 GC-MS library. All identified metabolites were manually inspected.

### Quantitative analysis and data integration.

Protein orthologues, lipids, or metabolites were considered significantly different with a *P* value of ≤0.05 using *t* test considering equal variance and two-tailed distribution. For comparative analyses, missing values were zero-filled with half of the smallest value of the data set. Proteins were clustered by the k-means method using Multi-Experiment Viewer (MeV, v4.9.0) (59), which was also used to build the heatmaps. Pathway analysis on different protein clusters was performed with DAVID (60), and specific pathways of interested were manually inspected with Vanted v2.1.1 (61). We have recently developed an R package called Rodin (https://github.com/PNNL-Comp-Mass-Spec/Rodin), to perform structural “lipid ontology” (LO) enrichment analysis. A web interface, Lipid Mini-On, was developed for non-R users (https://omicstools.pnnl.gov/shiny/lipid-mini-on/) (62). Briefly, this tool creates automatically LO bins based on the lipids naming and their inferred structure, and then it performs enrichment analysis using enrichment statistics to compare a query list to a Universe (Fisher exact test, EASE score, binomial test, or hypergeometric tests). In this study, a Fisher exact test was used to perform the enrichment analysis, and only the enrichment *P* values below 0.05 were considered significant.

### Data availability.

Proteomics data were deposited into Pride repository (www.ebi.ac.uk/pride) under accession numbers PXD013456 and PXD013457.
